# Homotaurine, a safe blood-brain barrier permeable GABA_A_-R-specific agonist, ameliorates disease in mouse models of multiple sclerosis

**DOI:** 10.1038/s41598-018-32733-3

**Published:** 2018-11-08

**Authors:** Jide Tian, Hoa Dang, Martin Wallner, Richard Olsen, Daniel L. Kaufman

**Affiliations:** Department of Molecular and Medical Pharmacology, University of California, Los Angeles, California USA

## Abstract

There is a need for treatments that can safely promote regulatory lymphocyte responses. T cells express GABA receptors (GABA_A_-Rs) and GABA administration can inhibit Th1-mediated processes such as type 1 diabetes and rheumatoid arthritis in mouse models. Whether GABA_A_-R agonists can also inhibit Th17-driven processes such as experimental autoimmune encephalomyelitis (EAE), a model of multiple sclerosis (MS), is an open question. GABA does not pass through the blood-brain barrier (BBB) making it ill-suited to inhibit the spreading of autoreactivity within the CNS. Homotaurine is a BBB-permeable amino acid that antagonizes amyloid fibril formation and was found to be safe but ineffective in long-term Alzheimer’s disease clinical trials. Homotaurine also acts as GABA_A_-R agonist with better pharmacokinetics than that of GABA. Working with both monophasic and relapsing-remitting mouse models of EAE, we show that oral administration of homotaurine can (1) enhance CD8^+^CD122^+^PD-1^+^ and CD4^+^Foxp3^+^ Treg, but not Breg, responses, (2) inhibit autoreactive Th17 and Th1 responses, and (3) effectively ameliorate ongoing disease. These observations demonstrate the potential of BBB-permeable GABA_A_-R agonists as a new class of treatment to enhance CD8^+^ and CD4^+^ Treg responses and limit Th17 and Th1-medaited inflammation in the CNS.

## Introduction

There is an unmet need for clinical treatments that can safely limit the progression of chronic inflammatory disorders. This is especially true for T-cell mediated autoimmune disorders such as type 1 diabetes (T1D), rheumatoid arthritis (RA), and multiple sclerosis (MS) in which inhibiting established autoimmune responses against the target tissue may require life-time treatment.

Rodent and human T cells express receptors for the nonprotein amino acid γ-aminobutyric acid (GABA)^1–5^. GABA is a commonly used neurotransmitter in the CNS and its neurobiological activities in brain development and function have been extensively studied. The immunobiological activities of GABA, however, are just beginning to be elucidated. There are two types of GABA-receptors (GABA-Rs) that are encoded by distinct gene families and their activation induces different pathways; GABA_A_-Rs are fast-acting chloride channels and GABA_B_-Rs are slow-acting G-protein coupled receptors^6,7^. Rodent and human T cells expresses functional GABA_A_-Rs but appear unresponsive to GABA_B_-R-specific agonists^1,3–5^. In mice, GABA administration limited delayed type hypersensitivity (DTH) responses and inhibited or reversed disease in models of T1D^1,2,5,8–10^, RA^11^, and limited inflammation and disease in type 2 diabetes models^12–14^. Studies of the mechanisms underlying those observations revealed that GABA treatment inhibited the development of autoreactive Th1 responses while also increasing CD4^+^ Tregs. These preclinical studies provided the basis for an ongoing clinical trial in which GABA is being given to individuals newly diagnosed with T1D (NCT02002130).

Whether this therapeutic approach can be extended to EAE and potentially to MS is unclear. First, while autoreactive Th1 cells are thought to be primary drivers of DTH responses, T1D, and RA disease progression, autoreactive Th17 responses are thought to play a major pathogenic role in EAE and MS^15,16^. Second, while DTH, T1D, and RA are mediated by peripheral autoimmune T cell responses, disease relapses in EAE and MS are thought be due to the spreading of T cell autoreactivity within the CNS^17–19^. Orally administered GABA, however, has little to no ability to pass through the BBB^20^, making it ill-suited to inhibit the spreading of T cell autoreactivities within the CNS. To circumvent that limitation, Steinman and colleagues^21^ showed that two clinically applicable BBB-permeable anti-seizure medications, topiramate and vigabatrin, that increase GABAergic tone, inhibited EAE. Topiramate, however, primarily affects other ion channels (e.g., sodium and calcium channels) and enzymes^22,23^ and vigabatrin does not bind to GABA-Rs but rather inhibits GABA transaminase^23^, and both drugs have multiple adverse effects. Other studies have shown that the anti-alcohol dependence drug acamprosate, which has structural similarities with GABA, can limit EAE^24^, but recent studies indicate that acamprosate does not act directly through GABA_A_-Rs^25,26^. Benzodiazepines and barbiturates are BBB-permeable GABA_A_-R positive allosteric modulators that can potentiate the opening of GABA_A_-R chloride channels, but only after a GABA_A_-R agonist opens the channel, and these drugs can be addictive. Accordingly, there is a need for BBB-permeable GABA_A_-R-specific agonists with excellent safety profiles that could potentially provide a new class of drugs for limiting inflammation in the CNS.

Homotaurine is a natural amino acid found in algae. Homotaurine emerged as a leading candidate from a screen for compounds that physically interfered with the ability of amyloid peptide to form fibrils *in vitro*^27,28^. Subsequent studies found that oral homotaurine can pass through the BBB and limit amyloid plaque deposition in the brain of transgenic mice that over-expressed human amyloid protein^27,28^. Based on these observations, homotaurine (also known as Tramiprosate or Alzhemed) was tested in a large double-blind randomized phase III clinical trial for its ability to slow cognitive loss over 1.5 years in patients with Alzheimer’s disease^29,30^. While homotaurine treatment was found to not slow cognitive decline, it had an excellent safety profile in this long-term study. Recently, it has become appreciated that homotaurine can also act as a GABA_A_-R-specific agonist. Homotaurine has >3-fold higher affinity for classical GABA_A_-Rs and a longer half-life than GABA in plasma (≈3 hours vs. 20 minutes for GABA after intravenous or intraperitoneal injection)^28,31–34^. Homotaurine’s pharmacokinetic properties, along with its safety record in humans, make it an appealing candidate for treating inflammation in the CNS. We therefore tested whether homotaurine could be repurposed for treating murine EAE. After finding homotaurine was effective in two different murine EAE models, we studied the treatment’s potential therapeutic mechanisms and observed, for the first time, that in addition to promoting CD4^+^Foxp3^+^ Treg responses, GABA_A_-R activation also enhances CD8^+^CD122^+^PD-1^+^ Treg responses and inhibits Th17 responses. Thus, GABA_A_-Rs are a new druggable target for enhancing CD8^+^ Treg responses and inhibiting Th17 responses in inflammation-related disorders.

## Results and Discussion

**Figure 1:**
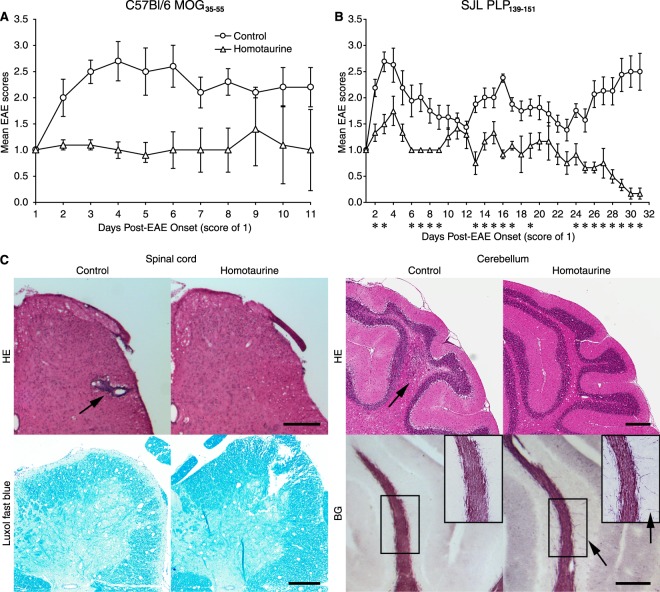
Homotaurine inhibits EAE progression after the onset of symptoms in both monophasic and relapsing-remitting mouse models of MS. (**A**) C57BL/6 mice were immunized with MOG_35–55_ and monitored daily for clinical signs of EAE as described in Methods. Eleven to thirteen days post-immunization all of the mice developed EAE. When the mice reached an EAE score of 1 they were randomized to receive plain water or water containing homotaurine (0.25 mg/ml). Graph shows mean EAE scores ± SEM for mice that received plain water (circles) and homotaurine (triangles) after EAE onset. N = 5 mice/group, overall p = 0.01, by Kruskal-Wallis analysis. (**B**) SJL mice were immunized with PLP_139–151_ and ten to twelve days later all of the mice develeoped clinical symptoms. When the mice reached an EAE score of 1 they were randomized to receive plain water or water containing homotaurine. Graph shows mean EAE scores ± SEM for mice that received plain water (circles) or homotaurine (triangles) continuously after EAE onset. N = 8 mice/group, *p < 0.05, overall p < 0.01. (**C**) Spinal cord (left panel) and cerebellum (right panel) in control and homotaurine-treated PLP_139–151_ immunized SJL mice. Twenty-seven days after treatment, adjacent sections were stained with hematoxylin and eosin (H &E, top row) and luxol fast blue or black gold (bottom row). Arrow in H&E stained sections indicates infiltrates. Inserts in black gold stained cerebellum images show boxed areas magnified 1.5X revealing greater myelination and fine myelin fibers in homotaurine-treated mice (arrows). Left panel scale bar = 1 mm, right panel scale bar = 250 µm.

We first tested the ability of homotaurine to limit disease progression in the chronic model of EAE that was induced by immunizing C57BL/6 mice with a myelin oligodendrocyte glycoprotein peptide (MOG_35–55_)^35^. Eleven to thirteen days after receiving the encephalitic regimen, all of the mice began to develop EAE. When an animal reached an EAE severity score of 1 it was randomly assigned to receive plain drinking water, or water containing homotaurine (0.25 mg/ml) for the rest of the observation period. This homotaurine dose was chosen based on our dosing studies of homotaurine’s ability to reverse disease in newly diabetic NOD mice (manuscript in preparation). Control and homotaurine-treated groups of mice drank similar amounts of water each day (≈4 ml/mouse/day). While the control group of mice quickly progressed to more severe EAE, the homotaurine treatment group of mice remained at low clinical score of EAE (Fig. 1A).

We next tested homotaurine treatment in a relapsing-remitting model of EAE that was induced by immunizing SJL mice with a proteolipid protein peptide (PLP_139–151_)^36^. Ten to 12 days after receiving the encephalitic regimen, all of the mice began to develop EAE. When an animal reached an EAE severity score of 1 it was randomly assigned to receive plain drinking water, or water containing homotaurine (0.25 mg/ml) for the rest of the study. Control mice displayed classical relapsing-remitting EAE. Homotaurine-treated mice exhibited a reduced mean EAE severity throughout the observation period compared to those given plain water, and eventually displayed almost complete remission (Fig. 1B). Histological analysis of their brains and spinal cords revealed reduced mononuclear cell infiltration and areas of myelin loss in the cerebellum and spinal cords of homotaurine-treated mice (Fig. 1C). Thus, treatment with homotaurine after the clinical onset of EAE inhibited disease progression in both monophasic and relapsing-remitting mouse models of MS. Previous studies using GABA to inhibit T1D and RA in mouse models could not distinguish the contributions of GABA_A_-Rs versus GABA_B_-Rs to those observations. Our results using homotaurine point directly to GABA_A_-R-mediated pathways as major components of GABA’s anti-inflammatory effects.

**Figure 2:**
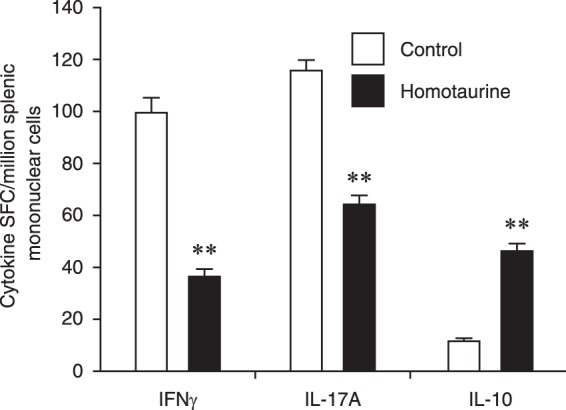
Homotaurine treatment at time of clinical EAE onset reduces the frequency of autoreactive IL-17A- and IFNγ-secreting T cell responses, while increasing IL-10-secreting responses. Mice were PLP_139–151_ immunized and after reaching an EAE score of 1, they received plain water or homotaurine for five days. Splenic T cells were isolated from individual mice and the percentages of IL-17A, IFNγ and IL-10 secreting T cells responding to PLP_139–151_ were measured by ELISPOT. Data shown are the mean SFC ± SEM of each group of mice (n = 5 per group) from two separate experiments. IL-4 responses to PLP_139–151_ were at background levels (data not shown). Wells containing cells from control or homotaurine-treated mice that were incubated with medium alone had 0–5 SFC. Pairwise comparisons were performed by 2-tailed Student’s t test. **p < 0.01 vs. the controls.

To begin to understand the basis for homotaurine’s therapeutic effects, we next examined homotaurine’s effects on lymphocytes since T cells are the major mediators of EAE and MS. SJL mice were immunized with PLP_139–151_ and 10 days later (when most mice had an EAE score of about 1), the mice were given plain water or water containing homotaurine (0.25 mg.ml) continuously for five days after which their splenic T cells were analyzed by ELISPOT. Consistent with previous studies of GABA treatment^2,5,8,10,11^, we observed that homotaurine treatment significantly decreased the frequency of splenic IFNγ (Th1) responses, but increased IL-10-secreting responses to PLP_139–151_ (Fig. 2). In addition, we show for the first time, that GABA_A_-R activation significantly reduces the frequency of PLP_139–151-_reactive IL-17A (Th17) spot-forming colonies (SFC) (Fig. 2). These IL-17A SFC represent antigen-specific activated/memory CD4^+^ T cells since peptide 13mers do not stimulate CD8^+^ T cells effectively, and it has been demonstrated in the PLP_139–151_/SJL model that IL-17 secreting SFC arise from CD4^+^ cells and not other cell types^37^. Homotaurine had no effect on Th2 frequencies (data not shown).

**Figure 3:**
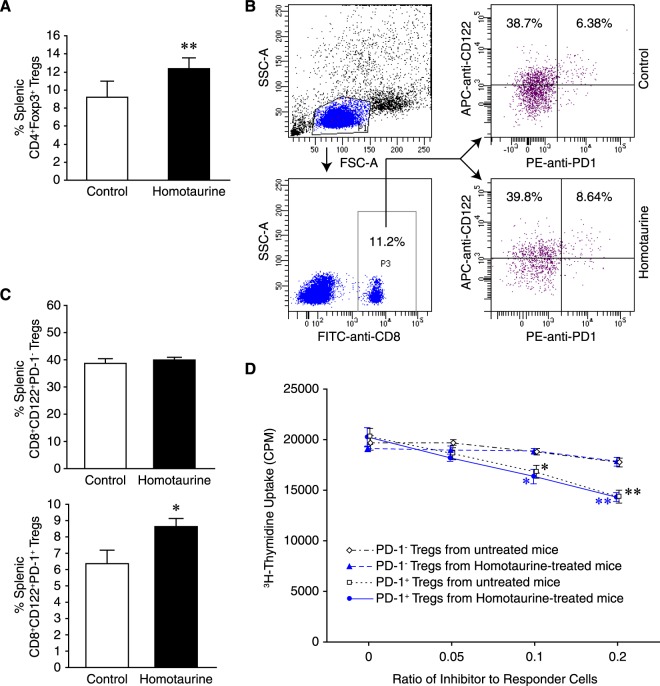
Homotaurine treatment enhances CD4^+^ and CD8^+^ Treg responses in mice. SJL mice were immunized with PLP_139–151_ in 50% CFA and when the mice developed EAE with a score of 1, they were randomized and provided with plain water and water containing 0.25 mg/ml homotaurine for five days. The percentages of splenic (**A**) CD4^+^Foxp3^+^, (**B,C**) CD8α^+^CD122^+^ and CD8α^+^CD122^+^PD-1^+^ Tregs were determined by flow cytometry. The cells were gated first on living lymphocytes and CD4^+^ or CD8^+^ and the percentages of CD4^+^Foxp3^+^, CD8α^+^CD122^+^PD-1^−^ and CD8α^+^CD122^+^PD-1^+^ Tregs were analyzed. Data are representative flow cytometry charts or expressed as the mean ± SEM of each group of mice (n = 5 per group) from two separate experiments. *p < 0.05, **p < 0.01 vs. the controls. (**D)** Splenic CD8^+^CD122^+^PD-1^+^ and CD8^+^CD122^+^PD-1^−^ Tregs were sorted by flow cytometry and mixed with lymph node mononuclear cells from PLP_139–151_-immunized SJL mice at the indicated ratios and assessed for PLP_139–151_-stimulated T cell proliferation. Data are expressed as mean ± SD of each group of cells. The cultures with responders in medium alone served the negative controls (with CPM of 850–940) and peptide-stimulated cultures without any inhibitor provided positive controls. Additional positive control cells were stimulated with PPD or anti-CD3 (with CPM of 20821–26759). *p < 0.05, **p < 0.01 vs. the peptide-stimulated positive controls.

GABA treatment can promote CD4^+^ Treg responses in mice^8,12^. It is unknown whether GABA_A_-R activation might also modulate CD8^+^CD122^+^PD-1^+^ and CD8^+^CD122^+^PD-1^−^ regulatory T cells or IL-10-secreting Bregs which can play crucial roles in inhibiting inflammation and T cell autoimmunity. We found that the percentages of splenic CD4^+^Foxp3^+^ Tregs (Fig. 3A) and CD8^+^CD122^+^PD-1^+^, but not CD8^+^CD122^+^PD-1^−^, Tregs (Fig. 3B,C) in the homotaurine-treated mice were significantly higher than in the controls. There was no significant difference in the frequency of splenic CD19^+^IL-10^+^ Bregs (data not shown).

To test the inhibitory function of CD8^+^CD122^+^PD-1^+^ and CD8^+^CD122^+^PD-1^−^ Tregs, SJL mice were immunized with PLP_139–151_ and 10 days later, they were randomized to receive homotaurine (0.25 mg/ml) through their drinking water, or plain water for the next six days. Their splenic CD8^+^CD122^+^PD-1^+^ and CD8^+^CD122^+^PD-1^−^ Tregs were sorted by flow cytometry and mixed with lymph node mononuclear cells from PLP_139–151_-immunized SJL mice for proliferation assays. Co-culture with CD8^+^CD122^+^PD-1^+^ Tregs, but not CD8^+^CD122^+^PD-1^−^ Tregs, from either homotaurine-treated or control (plain water)-treated mice significantly inhibited the proliferation of responders in a dose-dependent manner (Fig. 3D). The inhibitory effect of CD8^+^CD122^+^PD-1^+^ cells from homotaurine-treated mice was comparable to those from control mice, indicating that homotaurine treatment did not significantly alter their suppressive activity. These data, together with the increased frequency of CD4^+^Foxp3^+^ and CD8^+^CD122^+^PD-1^+^ Tregs in the homotaurine-treated mice indicate that CD4^+^Foxp3^+^ and CD8^+^CD122^+^PD-1^+^ Tregs play an important role in inhibiting the pathogenic T cell autoimmunity, contributing to the decreased severity of EAE.

Very few of the many drugs that can inhibit EAE successfully translated to the clinic. Factors that contribute to this failure include; (1) inherent differences between EAE and MS, (2) toxicity or side-effects in humans, and (3) supra-physiological doses were used in the EAE studies. In regard to those issues, human T cells express GABA_A_-Rs whose activation can limit PBMC inflammatory responses^3–5,38^, homotaurine appears to be safe for long-term human use, and using FDA guidelines (www.fda.gov/downloads/Drugs/…/Guidances/UCM078932.pdf) for scaling mouse doses to the equivalent human dosage, the homotaurine dose we used to inhibit EAE was 6–10-fold lower than that used in the Alzheimer’s disease clinical trials.

Murine and human T cells express relatively high levels of transcripts encoding the δ and ρ2 subunits of GABA_A_-Rs^2–5,38^. The δ and ρ2 subunits are of particular interest because thay are found in “extrasynaptic” GABA_A_-Rs that can be orders of magnitude more sensitive to GABA than the typical GABA_A_-Rs subtypes that are clustered in neuronal synapses^39–44^. These δ and /or ρ2 subunits may confer T cells with sensitivity to low levels of GABA and contribute to CNS “immunological privilege”. In this scenario, endogenous CNS GABA may help regulate the low-frequency CNS-reactive T cells that spontaneously activate under normal conditions, but it is insufficient to regulate the large number of autoreactive T cells that are experimentally activated in EAE. As we show here, administration of a BBB-permeable high-affinity GABA_A_-R agonist is capable of limiting CNS autoreactivity under those conditions. Additionally, homotaurine may have acted on antigen presenting cells (APC) such as macrophages, dendritic cells, and microglia that contribute to EAE pathogenesis^21,45,46^. These APC also express GABA_A_-Rs and GAB_A_A-R agonists have been shown to inhibit their inflammatory activity^11,21,47–49^.

In summary, our findings provide insights into the actions of GABA_A_-Rs on lymphocytes, as well as a proof-of-principle for a potential new class of drug treatment for enhancing Treg responses and limiting autoreactive Th17 cells. Our findings have potential for treating MS and may have broader applications for ameliorating other inflammation-related disorders of the CNS.

## Methods

### EAE induction and treatment

All experimental protocols were approved by the UCLA Animal Protection Committee and carried out in accordance with relevant guidelines. Nine to ten weeks old female C57BL/6 or SJL mice were obtained from the Jackson Laboratory and housed in a specific pathogene-free facility with free access of food and water. C57BL/6 mice were immunized subcutaneously with MOG_35–55_ (200 µg) in 50% IFA containing *Mycobacterium tuberculosis* H37R (5 mg/ml, Difco) in multiple sites near the base of their tail on day 1 and injected intraperitoneally with pertussis toxin (200 ng/mouse) on day 0 and 2. Individual SJL mice were immunized with PLP_139–151_ (100 µg/mouse), as described above, but without pertussis toxin injection. The mice were monitored for EAE onset daily: 0, no disease; 1, limp tail; 2, hind limb weakness; 3, complete hind limb paralysis; 4, quadriplegia; and 5, death. Mice that were in between the clear-cut gradations were scored intermediate in increments of 0.5. When the mice developed EAE with a score of 1 at 10–12 days post-immunization, they were randomized to receive plain water or water containing homotaurine (0.25 mg/ml (Sigma-Aldrich A76109), an optimal dose based on preliminary studies of T1D prevention in NOD mice).

### Histology

Mice were perfused with 4% paraformaldehyde in 0.1 M phosphate buffer, fixed overnight at 4 °C, cryoprotected and frozen. The brain and spinal cord cryostat sections (10 and 20 μm) were directly mounted on slides or collected into PBS with 0.06% NaN3. The sections were mounted onto Superfrost + slides and stained with H&E or Black Gold II. Some mouse brain and spinal cord tissues were paraffin-embedded after fixation and stained with H&E or luxol fast blue. The sections were imaged under a light microscope.

### ELISPOT assays for IFN, IL-17A, IL-10

SJL mice were immunized with 100 µg PLP_139–151_ in 50% IFA containing 5 mg/ml Mycobacterium tuberculosis H37R. When the mice developed EAE with a score of 1, the mice were randomized and received plain water or water containing 0.25 mg/ml homotaurine for 5 days. Their splenic mononuclear cells were isolated and the frequency of IFNγ, IL-17A, IL-4 and IL-10-secreting T cells responding to antigens were determined by ELISPOT as described previously^10^ with the addition that rat anti-mouse interleukin IL-17 (4 μg/ml, clone TC-11-18H10, BD Biosciences) and biotin-conjugated anti-IL-17 (5.0 μg/ml; Pharmingen, clone TC-11-8H4.1) were used as capture and detection antibodies for IL-17A, respectively. Briefly, splenic mononuclear cells (10^6^/well) were stimulated in duplicate with PLP139-151 (15 µg/ml), positive control PPD (5 µg/ml) or mouse serum albumin (50 µg/ml, Sigma) in HL-1 medium for 24 or 48 h. The spot forming colonies (SFC) in each well were counted in a blinded manner.

### FACS

When PLP_139–151_ immunized mice developed EAE with a score of 1, they were randomized to receive plain water or water containing 0.25 mg/ml homotaurine for 5 days. Splenic mononuclear cells (10^6^/tube) from the control and homotaurine-treated groups were treated with 1 µg anti-CD16/anti-CD32 and stained with FITC-anti-CD8α, APC-anti-CD122 and PE-anti-PD-1. After being washed, the frequency of CD8^+^CD122^+^PD1^−^ and CD8^+^CD122^+^PD-1^+^ regulatory T cells was determined by flow cytometry. In addition, splenic mononuclear cells (10^6^/tube) were stained with FITC-anti-CD4, fixed, permeabilized and intracellularly stained with PE-anti-Foxp3. The frequency of CD4^+^Foxp3^+^ Tregs was determined by flow cytometry. Moreover, splenic mononuclear cells (10^6^/tube) were stimulated with 50 nM PMA and 1 µM ionomycine (Sigma) for 2 h and in the presence of monensin for another 3 h. The cells were stained with FITC-anti-CD19, fixed, permeabilized and intracellularly stained with PE-anti-IL-10. The cells with isotype controls served as the negative controls. The frequency of CD19^+^IL-10^+^ Bregs was determined by flow cytometry.

### Functional assessment of CD8^+^ Treg suppressor activity

Female SJL mice were injected with 200 µg PLP_139–51_ in 50% CFA and nine days later, the mice were randomized and provided with, or without, homotaurine in their drinking water (0.25 mg/ml) for 6 days. The mice were then sacrificed, their splenic mononuclear cells were isolated, and CD8^+^ T cells were enriched by negative selection using the Majosort mouse CD8 T cell isolation kit (BioLegend, San Diego). The isolated CD8^+^ T cells were stained with FITC-anti-CD8, APC-anti-CD122 and PE-anti-PD-1 (BioLegend). The CD8^+^CD122^+^PD-1^+^ and CD8^+^CD122^+^PD-1^−^ Tregs were sorted by flow cytometry and used as inhibitors. In addition, SJL mice were immunized with 100 µg PLP_139–151_ in 50% CFA in their footpads and nine days later, their draining lymph node mononuclear cells were isolated and used as responders. To test the inhibitory function of CD8^+^ Tregs, lymph node mononuclear cells (1 × 10^5^ cells/well) were mixed in triplicate with CD8^+^CD12^+^PD-1^+^ or CD8^+^CD122^+^PD-1^−^ Tregs from either control or homotaurine-treated mice at ratios of 0.2, 0.1 or 0.05 (inhibitors to responders) and stimulated with PLP_139–151_ (20 µg/ml) for 96 hours. Some responders received medium alone (negative control) and other responders were stimulated with peptide antigen in the absence of inhibitory cells (positive control). Moreover, some responders were stimulated with PPD (10 µg/ml) or anti-CD3 (1 µg/ml) and served as additional positive controls. During the last 16-hour culture, individual wells of cells were exposed to 1 µci ^3^H-thymidine and the ^3^H-thymidine uptakes were measured by a β-counter.

### Statistics

EAE scores were evaluated using Kruskal-Wallis test. Pairwise comparisons were performed by 2-tailed Student’s t test. P < 0.05 was considered statistically significant.
